# Inconsistency in the Association Between Proton Pump Inhibitor Use and Dementia Risk: An Updated Meta-Analysis

**DOI:** 10.3390/brainsci16020159

**Published:** 2026-01-29

**Authors:** Tzu-Rong Peng, Hung-Hong Lin, Li-Jou Yang, Ta-Wei Wu

**Affiliations:** 1 Department of Pharmacy, Taipei Tzu Chi Hospital, Buddhist Tzu Chi Medical Foundation, New Taipei City 23142, Taiwan; tzu.rong@tzuchi.com.tw (T.-R.P.); zero8012@gmail.com (L.-J.Y.); 2 School of Pharmacy, College of Pharmacy, Taipei Medical University, Taipei 11031, Taiwan; 3 Department of Nursing, Tzu Chi University, Hualien 970374, Taiwan; 4 Department of Pharmacy, Chia-Nan University of Pharmacy and Science, Tainan 717301, Taiwan; h120076@ms13.hinet.net; 5 Department of Pharmacy and Master Program, Tajen University, Pingtung 90741, Taiwan

**Keywords:** proton pump inhibitors, dementia, Alzheimer’s disease, meta-analysis, cognitive decline, observational studies

## Abstract

Background: Proton pump inhibitors (PPIs) are commonly used to manage acid-related gastrointestinal conditions. Nevertheless, growing attention has been paid to their long-term safety, especially their possible link to dementia and Alzheimer’s disease (AD). Prior research has yielded inconsistent findings, underscoring the need for a comprehensive and current evaluation. Methods: A systematic search was conducted across PubMed, Embase (Ovid), and the Cochrane Library to identify relevant publications up to May 28, 2025, without language restrictions. Two investigators independently extracted study information and evaluated methodological quality as well as potential sources of bias. Eligible studies were observational in design and investigated the association between proton pump inhibitor (PPI) exposure and the risk of developing dementia compared with non-use. For the quantitative synthesis, pooled risk ratios (RRs) and corresponding confidence intervals were generated using a random-effects approach. Study Results: Eighteen studies, encompassing more than 6.3 million participants, met the inclusion criteria. The pooled estimate showed no statistically significant association between PPI use and overall dementia risk (RR = 1.14, 95% CI 0.98–1.33; I^2^ = 99%). However, significant heterogeneity and variable risk of bias—particularly due to confounding, exposure misclassification, and immortal time bias—limit certainty in these findings. Subgroup analyses revealed significantly elevated risks among individuals aged ≥65 years (RR = 1.21, 95% CI 1.01–1.46) and in studies from Asia (RR = 1.31, 95% CI 1.12–1.52) and Europe (RR = 1.32, 95% CI 1.10–1.59), suggesting possible population- or context-specific vulnerability. Conclusions: Our findings reveal a lack of consistent evidence supporting a link between PPI use and dementia risk, primarily due to significant heterogeneity among existing studies. While no robust overall association was demonstrated, significant subgroup signals in older adults and specific regions suggest that clinical uncertainty remains. Rather than indicating a direct causal risk, these results underscore the importance of prescribing stewardship. Clinicians should focus on appropriate prescribing, ensuring long-term PPI therapy is reserved for patients with a clear therapeutic justification and utilized for the shortest effective duration.

## 1. Background

The advent of proton pump inhibitors (PPIs) transformed therapeutic approaches to acid-related gastrointestinal diseases. Currently, they are extensively prescribed for disorders such as dyspepsia, gastroesophageal reflux disease, Barrett’s esophagus, Zollinger–Ellison syndrome, and Helicobacter pylori infection [1]. Long-term PPI use has become increasingly routine in clinical practice [2,3]. In many countries, the over-the-counter availability of these medications allows patients to use them without direct medical supervision [4,5]. Early investigations first highlighted a potential link between PPI use and dementia, reporting approximately a 40% increased risk among users [6,7], which has since drawn considerable attention.

In recent years, growing concern has emerged regarding the potential adverse effects of prolonged PPI use, particularly in relation to cognitive function and the risk of dementia [6,7,8,9]. Wijarnpreecha et al. [10] reported a possible association between PPI use and an increased risk of dementia, including Alzheimer’s disease (AD). However, several relevant studies published after the meta-analysis were excluded, producing inconsistent findings regarding this potential link [6,11,12,13].

Emerging evidence continues to explore how PPI use might influence cognitive health. While a secondary analysis of the ASPREE trial showed no association between PPIs and dementia incidence or cognitive decline among older adults, retrospective data from the ARIC study pointed toward a possible increase in risk with long-term exposure exceeding 4.4 years [14]. However, methodological limitations, such as reliance on International Classification of Diseases codes and lack of standardized cognitive assessments, have raised concerns about the validity of these findings. Expert reviews have cautioned against overinterpreting such associations, emphasizing the need for rigorous prospective data.

Given these discrepancies, the relationship between PPI use and risk of dementia or AD remains unclear. To address this gap, we conducted an updated meta-analysis that incorporated a broader range of studies to assess the potential association between PPI use and the development of dementia and Alzheimer’s more accurately.

## 2. Material and Methods

To achieve a standardized and transparent reporting process, we conducted this systematic review and meta-analysis in accordance with the 2020 PRISMA (Preferred Reporting Items for Systematic Reviews and Meta-Analyses) guidelines [15]. The complete PRISMA checklist detailing adherence to each reporting item is provided in Table S1. To enhance methodological rigor and reduce the risk of selective reporting, the study protocol was prospectively registered in the PROSPERO database (registration number: CRD420251076128), outlining the objectives, eligibility criteria, and planned analytical approaches before conducting the review.

### 2.1. Search Strategy

To identify relevant studies on PPI exposure and dementia risk, PubMed, EMBASE (Ovid), and the Cochrane Library were systematically searched from database inception through 28 May 2025. The search was conducted without language restrictions to capture a global body of evidence. As detailed in Supplementary Box S1, we employed a comprehensive strategy combining Medical Subject Headings (MeSH), controlled vocabulary, and Boolean operators.

Specifically, our search for PPI exposure included both class-level terms and specific pharmacological agents, such as omeprazole, pantoprazole, lansoprazole, rabeprazole, esomeprazole, dexrabeprazole, and dexlansoprazole, using both [mh] and [tiab] tags. We also incorporated mechanism-based descriptors like “proton-translocating ATPases/antagonists and inhibitors” to ensure all relevant biochemical literature was captured. For the outcome, we used a broad definition of “dementia” across all fields. Additionally, we manually screened the reference lists of all included studies and relevant reviews to identify any supplementary eligible publications.

### 2.2. Eligibility Criteria

Studies were considered eligible if they examined the relationship between proton pump inhibitor (PPI) use and the risk of developing dementia. Acceptable study designs included prospective cohort studies, nested case–control studies, case–cohort studies, and randomized controlled trials. To be included, studies needed to report sufficient data to calculate hazard ratios (HRs) with corresponding 95% confidence intervals (CIs). Only studies that reported dementia incidence, including Alzheimer’s disease, as a binary outcome were considered. All included studies had to be published in peer-reviewed journals with full-text availability. Studies were excluded if they were duplicate publications, conference abstracts, case reports, commentaries, proceedings, or experimental studies in animals.

### 2.3. Assessment of Study Quality

Two independent reviewers (L.J.Y. and H.H.L.) evaluated the methodological quality of the included studies. For non-randomized studies, the ROBINS-I tool was applied, covering seven domains of potential bias: confounding, participant selection, classification of interventions, deviations from intended interventions, missing data, outcome measurement, and selective reporting. Randomized controlled trials were appraised using the revised Cochrane risk-of-bias tool (ROB 2.0). Any disagreements between reviewers were resolved through discussion or, if necessary, consultation with a third reviewer. Each study was assigned an overall risk of bias, categorized as low, moderate, severe, or critical, according to predefined criteria. These assessments were then incorporated into the interpretation of the findings.

### 2.4. Data Extraction

Data were independently extracted by two reviewers using a standardized checklist. For each included study, the following information was collected: first author and publication year, country, data source, study design, participant age range, follow-up duration in years, sample size, outcomes assessed, ROBINS-I risk-of-bias evaluation, study objectives, and reported risk estimates with 95% confidence intervals (CIs). Any discrepancies between reviewers were resolved through discussion or, if needed, by consulting a third reviewer.

### 2.5. Statistical Analysis

All statistical procedures were conducted using RevMan (Cochrane Review Manager, version 5.4, Oxford, UK) and R software (version 4.4.3) with the metafor package. Effect measures reported as HRs, ORs, RRs, or IRRs were carefully handled to avoid inappropriate pooling. Under the rare-outcome assumption, HRs and ORs were converted to log-RRs for a standard effect scale; conversion methods and assumptions are transparently documented. When conversion was not appropriate, study designs were analyzed separately (design-specific meta-analyses).

Pooled estimates were calculated using a random-effects model and presented as RRs with 95% confidence intervals (CIs). Between-study heterogeneity was assessed with the chi-square (χ^2^) test and the I^2^ statistic. To explore sources of heterogeneity and evaluate the robustness of the results, we conducted subgroup analyses by age, geographic region, and study design, as well as sensitivity analyses that included leave-one-out procedures and the exclusion of studies with a serious or critical risk of bias. Publication bias was assessed visually with funnel plots and quantitatively using Egger’s regression test. Statistical significance was defined as a two-sided *p*-value < 0.05.

## 3. Results

### 3.1. Search Strategy and Description of Included Studies

**Figure 1:**
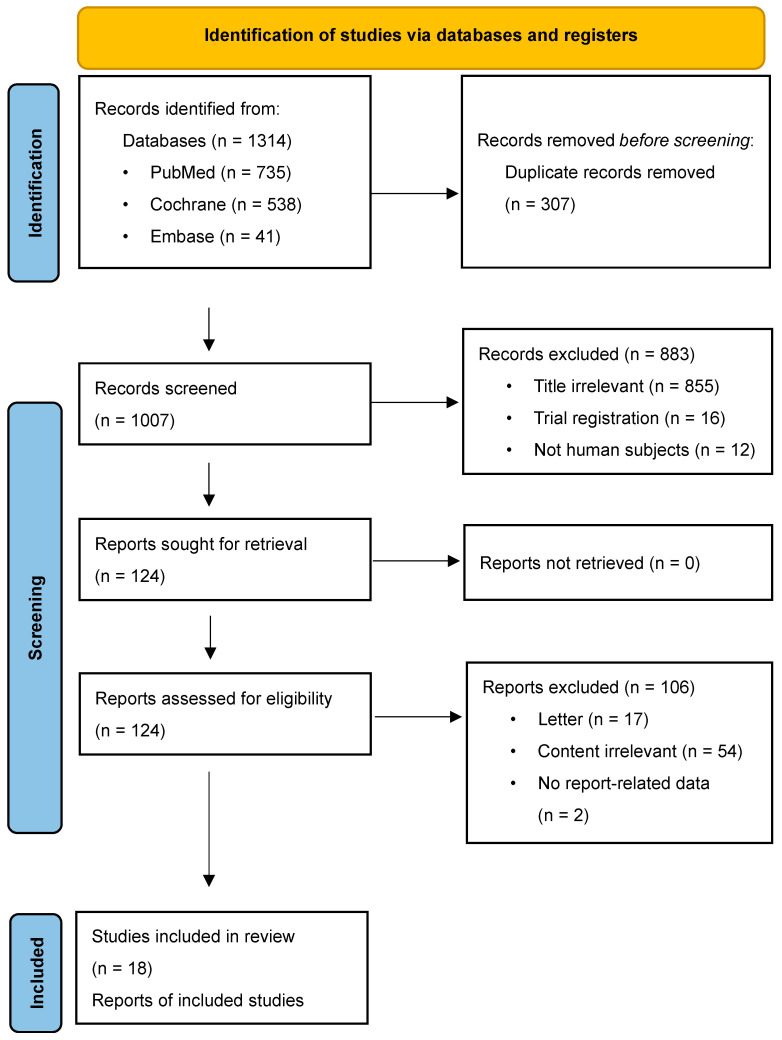
PRISMA flowchart illustrating the process of study identification, screening, eligibility assessment, and final inclusion.

Figure 1 presents a flow diagram outlining the identification and selection of studies included in the meta-analysis. The initial database search retrieved 1314 records, of which 307 duplicates were removed. Following title and abstract screening, 883 records were excluded. A total of 124 full-text articles were then assessed for eligibility according to predefined inclusion criteria, resulting in 18 studies being included in the final meta-analysis.

**Figure 2:**
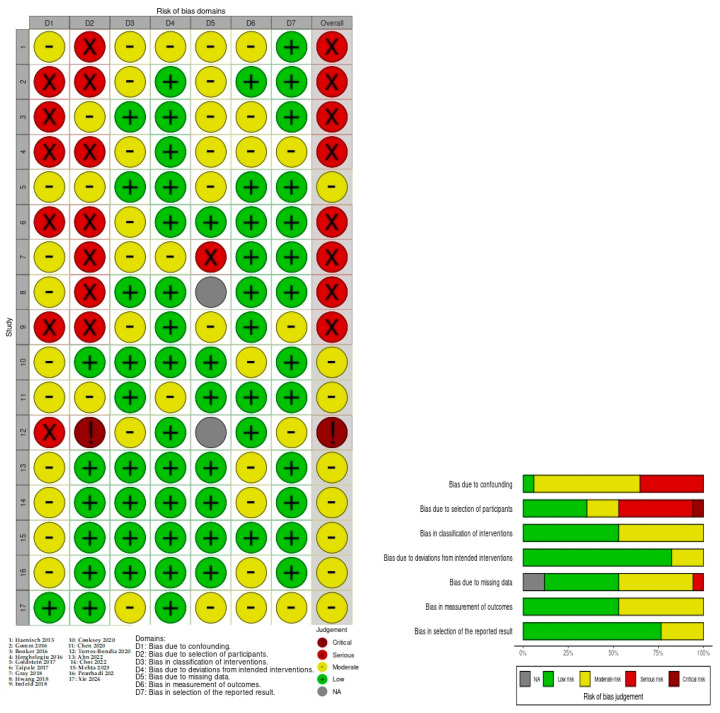
Traffic Light Plot of Risk of Bias for Cohort Studies [6,7,11,12,16,17,18,19,20,21,22,23,24,25,26,27,28] and Overall Summary of Each Domain.

Table 1 summarizes the key characteristics of the studies included in this meta-analysis. Sample sizes of the eligible studies ranged from 3076 to 2,698,176 participants, with a cumulative total of 6,325,898 individuals. Four studies were conducted in Germany, three in the United Kingdom, and the others in Denmark, Finland, Georgia, South Korea, the United States, Spain, and Taiwan. The methodological quality of the included studies was evaluated using the ROBINS-I tool. Risk of bias assessment using the ROBINS-I tool is visualized in Figure 2. Of the included studies, Torres-Bondia et al. [16] was judged to be at critical risk of bias due to participant selection issues. Several studies were rated as serious risk, primarily driven by confounding (D1) and participant selection (D2). The remaining studies, including the large-scale analysis by Pourhadi et al. [17] and Mehta et al. [14], were rated as moderate or low risk overall. One study was an RCT [18], which was evaluated using the ROB 2.0 and assessed to have a low overall risk of bias, with its five domains rated as low, low, low, unclear, and low risk, respectively. This finding supports the reliability of the pooled estimates used in this meta-analysis. A detailed assessment of the risk of bias for each study using the ROBINS-I tool is presented in Figure 2.

### 3.2. Association Between PPI and Dementia or AD

**Figure 3:**
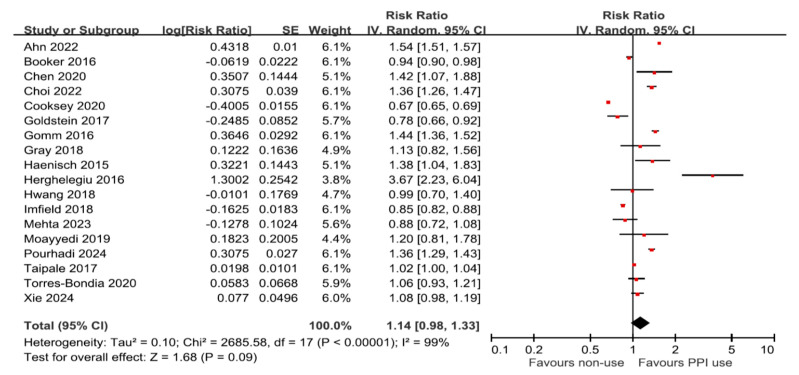
Random-effects meta-analysis forest plot summarizing the effect of proton pump inhibitor use on the risk of developing all-cause dementia, with individual study estimates and pooled risk ratios displayed.

**Figure 4:**
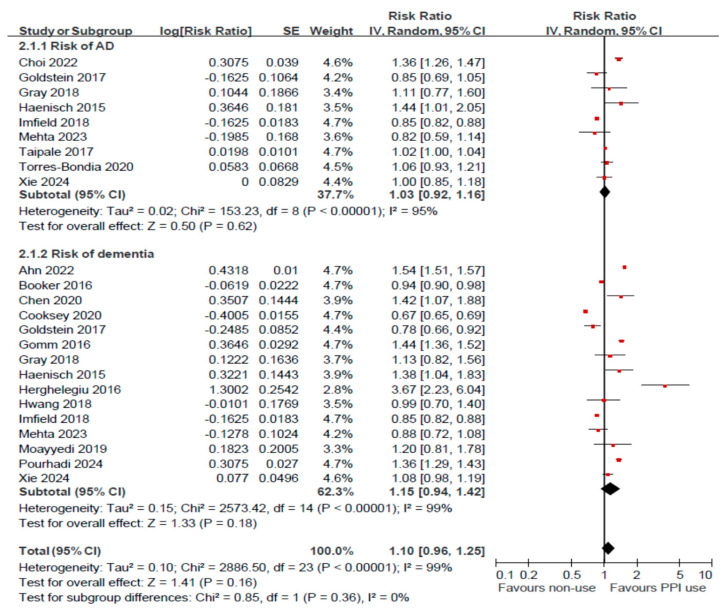
Forest plot of a random-effects meta-analysis assessing the risk of different types of dementia among proton pump inhibitor users compared to non-users.

Eighteen studies assessed the association between PPI use and the risk of all-cause dementia. The pooled analysis using a random-effects model showed no statistically significant association between PPI use and overall dementia risk (RR = 1.14, 95% CI: 0.98–1.33; I^2^ = 99%; *p* = 0.09; Figure 3). In a subgroup analysis, 15 studies specifically evaluated the association between PPI use and the risk of dementia, while nine studies focused on AD. Consistently, no significant associations were found in either subgroup: for AD (RR = 1.03, 95% CI: 0.92–1.16; I^2^ = 95%; *p* = 0.62) and for dementia (RR = 1.10, 95% CI: 0.96–1.25; I^2^ = 99%; *p* = 0.16; Figure 4). These results reflect a lack of consistent evidence supporting a significant association between PPI use and the risk of all-cause dementia or AD.

### 3.3. Subgroup Analyses

Stratified analyses based on study quality, design, baseline age, geographic region, and assessed effects revealed significant variations across subgroups (Table 2). Notably, a statistically significant positive association was observed in individuals aged ≥65 years (RR = 1.21, 95% CI: 1.01–1.46), as well as in studies conducted in Asia (RR = 1.31, 95% CI: 1.12–1.52) and Europe (RR = 1.32, 95% CI: 1.10–1.59). In contrast, studies from the United States showed a non-significant protective trend (RR = 0.87, 95% CI: 0.74–1.02). Moreover, the definition of exposure appeared to affect results: the “starting and adhering” effect (RR = 1.26, 95% CI: 0.98–1.62) suggested a higher risk compared with the “assignment” effect (RR = 0.99, 95% CI: 0.89–1.10).

### 3.4. Subgroup Analysis of Older Adults (≥65 Years) by Region

In a targeted subgroup analysis of older adults aged *≥* 65 years, the association between PPI use and dementia risk was evaluated across different geographic regions. For this age group, a significant risk was observed in the Asian cohort (RR = 1.42, 95% CI: 1.07–1.88, *p* = 0.015), while the European subgroup showed a non-significant trend (RR = 1.24, 95% CI: 0.89–1.72, *p* = 0.203) with extreme heterogeneity (I^2^ = 97.8%). To determine if these regional differences were statistically significant within the older population, a formal interaction test was performed. The test for subgroup differences yielded a non-significant result (*p* = 0.53), indicating that the observed geographic variations in the ≥65 age group did not reach statistical significance when accounting for within-region variability. Detailed results of this analysis are provided in Supplementary Table S2.

### 3.5. Sensitivity Analysis

**Figure 5:**
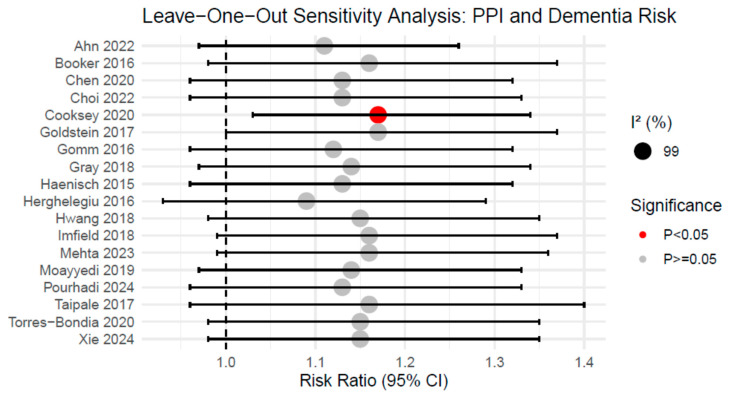
Results of Sensitivity Analysis for the risk of dementia and PPI use.

A sensitivity analysis was conducted using the leave-one-out approach to assess the robustness of the meta-analysis findings. The pooled RRs ranged from 1.09 to 1.17 across iterations, with corresponding 95% CIs of 0.93 to 1.40 for the lower and upper bounds, respectively (Figure 5) (Supplementary Table S3). Heterogeneity remained consistently high (I^2^ = 99%), regardless of which study was excluded. Notably, while most exclusions did not alter the overall significance, the exclusion of the study by Cooksey et al. yielded a statistically significant pooled estimate (RR = 1.17, 95% CI: 1.03–1.34, *p* = 0.02). This indicates that the findings exhibit a degree of fragility and are sensitive to the inclusion of specific large-scale datasets. Consequently, rather than demonstrating a definitive absence of risk, these results reflect current clinical uncertainty and underscore the need for a cautious interpretation of the evidence.

### 3.6. Publication Bias

**Figure 6:**
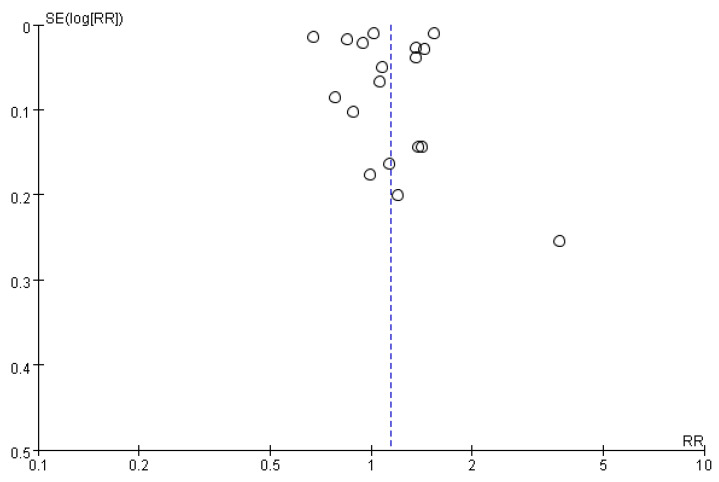
Funnel plot of the association between proton pump inhibitor (PPI) use and dementia risk.

In this review, visual inspection of the funnel plot revealed a slight asymmetry in the distribution of cognitive function (Figure 6). Publication bias was assessed using the Begg and Mazumdar tests. The Begg’s test yielded a Kendall’s tau of 0.1373, with a *p*-value of 0.4543, indicating no statistically significant evidence of publication bias. The Egger’s test yielded a borderline result, with a regression intercept of −0.0180 (95% CI: −0.2254 to 0.1894) and a *p*-value of 0.0618, indicating a trend toward funnel plot asymmetry but not reaching conventional statistical significance. Overall, these results did not provide strong evidence of publication bias in the included studies.

### 3.7. Quality of Evidence (GRADE Assessment)

The certainty of evidence for each primary and secondary outcome was evaluated using the GRADE (Grading of Recommendations, Assessment, Development, and Evaluations) framework and is summarized in Table 3. Due to the observational nature of the included studies, the initial certainty was rated as low, with further downgrades applied. The overall dementia risk and Alzheimer’s disease risk were rated as Very Low certainty, primarily due to extreme heterogeneity (*I*^2^ = 99%) and serious risk of bias in confounding and exposure misclassification. Subgroup findings for age ≥65 and specific regions were rated as Low certainty, as these significant signals remain sensitive to the high between-study variability inherent in observational data.

## 4. Discussion

This updated meta-analysis, encompassing 18 observational studies and over 6.3 million participants, found a lack of consistent evidence supporting a significant association between PPI use and the risk of all-cause dementia or AD. While these findings align with several high-quality recent reports, such as the ASPREE trial post-hoc analysis by Mehta et al. [27] and the large-scale cohort by Pourhadi et al. [17], the results must be interpreted with substantial caution due to the extreme heterogeneity (I^2^ = 99%) and the Very Low to Low certainty of evidence as assessed by the GRADE framework.

A primary challenge in interpreting these findings is the inclusion of studies with serious or critical risks of bias. Our ROBINS-I assessment identified significant concerns regarding confounding, exposure misclassification, and immortal time bias in several large-scale cohorts. For instance, the reliance on administrative ICD codes for diagnosis and the potential for immortal time bias—where the period before the first prescription is misclassified—can artificially attenuate risk estimates and mask true associations, as noted in previous critical reviews [29]. Combining these high-risk studies with more rigorous cohorts introduces substantial noise, contributing to the observed inconsistency.

The fragility of the overall estimate was further highlighted by our sensitivity analysis. Notably, the exclusion of a single influential study by Cooksey et al. [24] rendered the association between PPI use and dementia risk statistically significant (RR = 1.17, 95% CI: 1.03–1.34). This sensitivity indicates that the “null” result is not sufficiently robust to draw a definitive conclusion of safety. Rather than demonstrating a definitive absence of association, our results reflect current clinical uncertainty and underscore the importance of prescribing stewardship. Clinicians should ensure that long-term PPI therapy is reserved for appropriate indications and regularly reviewed, prioritizing patient safety amidst the lack of consensus in current observational data.

In contrast, earlier meta-analyses, such as that by Wijarnpreecha et al., suggested a potential increase in dementia risk; however, these analyses were constrained by smaller sample sizes, shorter follow-up durations, and a higher susceptibility to bias [10]. Notably, our subgroup analyses revealed statistically significant increases in dementia risk among individuals aged ≥65 years (RR = 1.21, 95% CI: 1.01–1.46) and in studies conducted in Asia (RR = 1.31, 95% CI: 1.12–1.52) and Europe (RR = 1.32, 95% CI: 1.10–1.59). These findings may indicate genuine signals of harm in specific populations, potentially driven by regional prescribing practices, genetic predispositions, access to healthcare, or environmental exposures. Conversely, studies from the United States demonstrated a non-significant protective trend (RR = 0.87, 95% CI: 0.74–1.02), suggesting that contextual or methodological factors may shape observed associations. This divergence may be further explained by the methodological distinction between “assignment” and “starting and adhering” exposure effects. In most included observational studies, exposure is defined by “assignment” based on prescription records. However, this may not fully capture the “starting and adhering” behavior, especially in regions with high over-the-counter (OTC) PPI availability, such as the United States. In such contexts, individuals in the control group may self-medicate with OTC PPIs, leading to significant exposure misclassification. This bias typically shifts the results toward the null, potentially diluting the observed risk and contributing to the non-significant findings in the U.S. cohort.

The Very Low to Low certainty of the evidence, as assessed by the GRADE framework, reinforces our finding of a lack of consistent evidence rather than a definitive proof of safety. These results reflect current clinical uncertainty and the discrepancy between various study designs, population characteristics, and real-world observational conditions. Consequently, the high heterogeneity and diagnostic variability underscore the fragility of the overall estimate and the need for cautious interpretation.

The observed geographical discrepancies may be attributed to several factors. First, diagnostic variability and differences in healthcare infrastructure—such as the reliance on administrative ICD codes—may shape the accuracy of dementia detection across regions. Second, the high prevalence of OTC PPI use in certain regions, particularly the U.S., can lead to significant exposure misclassification. Regarding geographic distribution, while our subgroup analysis of older adults (≥65 years) showed a higher risk estimate in the Asian population (RR 1.42, 95% CI 1.07–1.88), the formal interaction test yielded a non-significant result (*p* = 0.53). This suggests that the observed regional differences may not be statistically robust in this age group, and that advanced age itself could be a more critical confounding factor than geographic location. The high heterogeneity observed across regions may also stem from variations in healthcare infrastructure and the availability of over-the-counter PPIs, rather than inherent ethnic susceptibility. Third, genetic predispositions, such as polymorphisms in the CYP2C19 enzyme which affect PPI metabolism, may vary by ethnicity and influence drug-related neurotoxicity. Finally, regional differences in prescribing stewardship and clinical guidelines likely contribute to the divergent findings, further underscoring the fragility of a single global estimate. Furthermore, it is essential to more clearly distinguish between all-cause dementia and AD as separate clinical outcomes. In this meta-analysis, while we evaluated these outcomes independently where data permitted, we must acknowledge that many included cohorts used administrative claims data or ICD codes that may overlap or lack the precision to differentiate specific neurodegenerative etiologies. The diagnostic criteria varied significantly across studies, ranging from standardized protocols like ICD-10 and DSM-IV to the use of billing codes or specialized neurological assessments. This variability in diagnostic rigor is a primary contributor to the extreme heterogeneity (I^2^ = 99%) observed in our results. The lack of biomarker-confirmed cases (e.g., amyloid PET or CSF analysis) in most large-scale observational studies further limits the ability to isolate the specific impact of PPIs on Alzheimer’s pathology. These discrepancies reinforce our GRADE assessment of Very Low to Low certainty (Table 3), as the evidence is based on heterogeneous definitions that may mask true disease-specific associations. While several biological mechanisms have been proposed to explain how PPIs might theoretically impact cognitive health—including amyloid-beta peptide accumulation [10] and Vitamin B12 malabsorption [6,7], and gut–brain axis alterations—our meta-analysis highlights a profound lack of consistent evidence across the current literature. Although the pooled estimate did not reach statistical significance, this finding must be interpreted with caution due to the extreme heterogeneity observed (I^2^ = 99%). Such high inconsistency suggests that the divergent results in epidemiological studies may be driven by variations in study design, population characteristics, or residual confounding inherent in observational data. Consequently, rather than demonstrating a definitive absence of association, our results reflect current clinical uncertainty and the discrepancy between preclinical models and real-world observational evidence.

The role of the APOE epsilon 4 allele as a potential moderator of PPI-associated neurotoxicity warrants attention. Theoretically, PPIs may impair amyloid-beta clearance—a process already compromised in APOE epsilon 4 carriers—by inhibiting the V-type ATPase and potentially increasing Aβ production. However, clinical evidence remains inconsistent. While preclinical models suggest increased vulnerability in these individuals, high-quality prospective data, such as the post-hoc analysis of the ASPREE trial by Mehta et al. [27], failed to confirm a significant interaction between PPI use and APOE status in older adults. This discrepancy underscores the current clinical uncertainty and highlights the need for future studies to incorporate genetic stratification to clarify genotype-medication interactions. Furthermore, as reflected in our subgroup analysis of older adults (≥65 years), the lack of a statistically significant interaction between geographic regions (*p* = 0.53) suggests that genetic factors, such as APOE or CYP2C19 polymorphisms, may be masked by the extreme heterogeneity observed in real-world observational data.

The relationship between PPI use and dementia risk is particularly complex in the oldest old, where the compounding effects of polypharmacy and age-related physiological changes are most prominent. Older adults frequently possess multiple comorbidities, leading to the concurrent use of various medications that may interact with PPIs via the cytochrome P450 system or exacerbate cognitive frailty. Our analysis suggests that the risk profile may not be uniform across the older age spectrum. Studies focusing on the “very old” (e.g., aged 80–90 years) have occasionally reported stronger risk signals, potentially due to reduced physiological clearance and increased blood–brain barrier permeability in advanced age. In contrast, younger seniors (e.g., aged 60–69 years) often show non-significant associations which may be attributed to a higher cognitive reserve that masks potential neurotoxic effects. However, our subgroup interaction test for the ≥65 population yielded a non-significant result (*p* = 0.53), indicating that these age-specific differences are often obscured by extreme between-study heterogeneity. Consequently, the observed associations in the 80–90-year-old group might be driven by the complexities of polypharmacy and “confounding by frailty” rather than a direct causal effect of PPIs. Regarding the impact of gender, our meta-analysis did not identify a consistent or statistically significant difference in dementia risk between male and female PPI users. Most included studies treated sex as a baseline covariate for adjustment rather than a primary stratification factor. While some earlier reports suggested potential variations, high-quality prospective data, such as the ASPREE trial, confirmed that the lack of association between PPI use and cognitive decline was consistent across both genders. Furthermore, older women often experience higher rates of polypharmacy and a longer lifespan, which may introduce “confounding by frailty” or survival bias in observational cohorts. Consequently, current evidence does not support gender-specific prescribing guidelines, reinforcing that prescribing stewardship should be applied equally to both men and women.

From a clinical perspective, these findings suggest potential signals of safety but must be interpreted with caution, emphasizing current clinical uncertainty. Rather than justifying a complete avoidance of these effective medications, our results reinforce the value of prescribing stewardship and the necessity of regularly reviewing the need for continued therapy. Current guidelines emphasize that PPI use should be guided by established clinical indications—such as erosive esophagitis, Barrett’s esophagus, or the prevention of NSAID-induced ulcers—and clinicians should aim for judicious prescribing at the lowest effective dose for the shortest required duration. This approach ensures patient safety across all domains while minimizing potential long-term risks in vulnerable populations, such as older adults.

The conclusion of a ‘lack of consistent evidence’ must be interpreted with caution. Our sensitivity analysis demonstrated that omitting a single large-scale study (Cooksey et al.) [24] rendered the association between PPI use and dementia risk statistically significant (RR = 1.17, 95% CI: 1.03–1.34). This shift underscores the fragility of the overall estimate and indicates that the primary result is highly dependent on specific datasets. Rather than reflecting a lack of consistent evidence across the current literature, these results reflect current clinical uncertainty and highlight the discrepancy between various real-world observational datasets. Such sensitivity, combined with the extreme heterogeneity observed (I^2^ = 99%), confirms that the evidence is not sufficiently robust to draw a definitive conclusion of safety, further underscoring the need for prescribing stewardship and appropriate therapeutic justification.

Importantly, several studies were rated as having severe or critical risks of bias in key domains of the ROBINS-I assessment (Figure 2), particularly due to confounding, exposure misclassification, and immortal time bias. These biases could have attenuated or inflated the estimated associations. For example, inadequate adjustment for comorbidities or concurrent medications may obscure true associations, while misclassification of intermittent or over-the-counter PPI use may dilute exposure effects. The presence of such biases substantially limits the certainty of the pooled findings and reinforces the need for cautious interpretation.

This meta-analysis has several limitations that should be acknowledged. First, most included studies were observational in design, which restricts definitive causal inference and may be subject to residual confounding or reverse causality. Second, the substantial heterogeneity (I^2^ = 99%) limits the interpretability of the summary effect, even with random-effects modeling. Thirdly, it is important to acknowledge the increased risk of false-positive findings arising from the multiple subgroup analyses conducted. Although the significant associations observed in older adults and specific geographic regions provide valuable clinical signals, these results should be interpreted as hypothesis-generating rather than confirmatory due to the lack of formal adjustment for multiple testing. Furthermore, while we conducted tests for publication bias and found no statistically significant evidence, it is important to note that the power to detect such bias is significantly limited in the presence of extreme heterogeneity (I^2^ = 99%). The vast differences in study designs, populations, and follow-up durations across the 18 included studies may mask potential asymmetries in the funnel plot6. Therefore, the influence of unpublished negative results or small-study effects cannot be entirely ruled out, and this limitation should be considered when interpreting the overall lack of consistent evidence. Finally, as reflected in our GRADE assessment, the overall certainty of evidence remains Very Low to Low, primarily due to methodological biases such as immortal time bias and exposure misclassification in several included cohorts.

Future studies should aim to reduce methodological variability by adopting standardized definitions of PPI exposure and dementia outcomes, as well as incorporating more extended follow-up periods and rigorous control of confounders. Investigating potential biological mechanisms underlying PPI use and cognitive outcomes will also be essential to clarify whether the observed associations are causal or spurious.

## 5. Conclusions

In conclusion, this systematic review and meta-analysis provides no clear or consistent evidence that PPI use significantly increases the risk of dementia or AD. However, the extreme heterogeneity highlights ongoing clinical uncertainty. Instead of a complete avoidance, we recommend a prescribing stewardship approach for aged patients: for administration, clinicians should prioritize short-term, goal-oriented therapy, and for patients requiring long-term use, consider on-demand or intermittent therapy to minimize cumulative exposure; regarding dosage, always utilize the lowest effective dose, which should be carefully calibrated in specific populations (e.g., Asian cohorts) due to metabolic variations such as CYP2C19; and for follow-up, conduct regular clinical reviews every 3 to 6 months to re-evaluate the necessity of the medication and consider prescribing if symptoms are controlled. Until further harmonized prospective data are available, clinicians should focus on these appropriate prescribing practices to ensure patient safety while minimizing unnecessary long-term exposure.

## Figures and Tables

**Table 1:** Overview of characteristics for included studies.

Study Author(s) and Year	Geographic Location	Dataset Used	Type of Study	Age Range	Follow-Up (Years)	Sample Size		Outcome Measured		ROBINS-I Aim of the Study	Funders
						Cohort Size	Cases	Alzheimer’s Disease	Dementia		
Haenisch, 2015 [6]	Germany	Study on ageing, cognition, and dementia in primary care patients	Population cohort	>75	Up to 6	3076	431	HR: 1.44 (1.01, 2.06)	HR: 1.38 (1.04, 1.83)	Starting and adhering	German Federal Ministry of Education and Research
Gomm, 2016 [7]	Germany	AOK Germany	Population cohort	>75	Up to 7	73,679	29,510	–	HR: 1.44 (1.36, 1.52)	Starting and adhering	Not explicitly stated; utilize AOK Germany claims data
Booker, 2016 [19]	Germany	The Disease Analyzer database (IMS Health)	Case–control	70–90	10	23,912	11,956	–	OR: 0.94 (0.90, 0.97)	Starting and adhering	Not explicitly stated
Herghelegiu, 2016 [20]	Romania	“Ana Aslan” National Institute of Geriatrics and Gerontology	Case–control	>65	3	148	26	–	OR: 3.67 (2.23, 19.15)	Starting and adhering	Not explicitly stated
Goldstein, 2017 [11]	United States/Georgia	National Alzheimer’s Coordinating Center database from 33 ADCs from September 2005 through September 2015	Observational, longitudinal	≥50	NR	10,486	3082	HR: 0.85 (0.69, 0.98)	HR: 0.78 (0.66, 0.93)	Starting and adhering	National Institute on Aging
Taipale, 2017 [21]	Finland	Cases: Finnish medication and Alzheimer’s disease (MEDALZ) study controls: Finnish nationwide healthcare registries	Nested case–control	>60	Up to 16	353,576	70,718	OR: 1.02 (1.00, 1.04)	–	Assignment	Social Insurance Institution of Finland
Gray, 2018 [12]	USA	Adult changes in thought study	Population cohort	>65	10	3484	827	HR: 1.11 (0.77, 1.61)	HR: 1.13 (0.82, 1.56)	Starting and adhering	National Institute on Aging
Hwang, 2018 [22]	South Korea	National Health Insurance Corporation	Population cohort	>60	Up to 7	70,033	1297	–	HR: 0.99 (0.70, 1.39)	Assignment	Not explicitly stated
Imfeld, 2018 [23]	UK	Clinical practice research datalink	Nested case–control	>65	Up to 15	82,058	41,029	OR: 0.85 (0.82, 0.89)	–	Assignment	Not explicitly stated
Moayyedi, 2019 [18]	33 countries	Multicenter	Multicenter double-masked randomized controlled trial	≥65	3 years	8791	8807	–	OR: 1.2 (0.81, 1.78)	Assignment	Bayer AG
Cooksey, 2020 [24]	UK	Data were obtained from the Secure Anonymised Information Linkage (SAIL) databank	Population cohort	>55	Up to 15	315,078	37,148	–	HR: 0.67 (0.65, 0.67)	Starting and adhering	Health and Care Research Wales
Chen, 2020 [25]	Taiwan	Longitudinal Health Insurance Database 2000 (LHID)	Population cohort	>65	Up to 14	18,800	3746	–	HR: 1.42 (1.07, 1.84)	Starting and adhering	Ministry of Education (Taiwan), China Medical University, and Chang Gung Memorial Hospital
Torres-Bondia, 2020 [16]	Spain	Catalan health service (CatSalut) system, Catalan Institute of Health (ICS)	Population cohort	>45	Up to 14	135,722	1135	OR: 1.06 (0.93, 1.21)	–	Assignment	Institut de Recerca Biomèdica de Lleida
Ahn, 2022 [26]	Germany	AOK Bayern	Population cohort	>40	Up to 10 years	2,698,176	56,576	–	HR: 1.54 (1.51, 1.580)	Starting and adhering	Innovation Committee at the Federal Joint Committee, Germany.
Choi, 2022 [27]	South Korea	Korean National Health Insurance Service-Health Screening Cohort	Population cohort	>60	Up to 10 years	86,125	17,225	OR: 1.36 (1.26, 1.46)	–	Starting and adhering	National Research Foundation of Korea
Mehta, 2023 [14]	United States and Australia	post-hoc analysis of ASPirin in Reducing Events in the Elderly (ASPREE)	Randomized trial	≥65	Up to 5	18,934	4667	HR: 0.82 (0.59, 1.14)	HR: 0.88 (0.72, 1.08)	Assignment	National Institute on Aging and the National Health and Medical Research Council of Australia
Pourhadi, 2024 [17]	Denmark	Nationwide Danish registers	Population cohort	60–75	Up to 19 years	1,983,785	99,384	–	IRR: 1.36 (1.29, 1.43)	Starting and adhering	Danish Alzheimer Foundation (Alzheimer-forskningsfonden)
Xie, 2024 [28]	UK Biobank	UK Biobank	Case–control	NR	NR	440,035	16,241	OR: 1.00 (0.85, 1.19)	OR: 1.08 (0.98, 1.18)	Assignment	Basic and Applied Basic Research Foundation of Guangdong Province and Natural Science Foundation of Guangdong Province

**Table 2:** Subgroup meta-analyses comparing dementia risk between PPI users and non-users.

Outcomes	Number of Studies	Summary Risk Ratio (95% CI)	Results of the Heterogeneity Test	
			*I* ^2^	*p* Value
Study design				
Prospective cohort	11	1.17 (0.90 to 1.52)	100%	0.24
Case–control study	5	1.02 (0.91 to 1.14)	96%	0.70
Minimum age at baseline				
Age ≥ 65	9	1.21 (1.01 to 1.46)	97%	0.04
Age < 65	9	1.07 (0.84 to 1.34)	100%	0.59
Follow-up (years)				
≥10 years	10	1.10 (0.89 to 1.35)	100%	0.38
<10 years	8	1.20 (0.97 to 1.49)	93%	0.10
Geographic region				
Asia	3	1.31 (1.12 to 1.52)	38%	0.0007
Europe	8	1.32 (1.10 to 1.59)	99%	0.003
United States	6	0.87 (0.74 to 1.02)	97%	0.09
Assessed effect				
Assignment effect	7	0.99 (0.89 to 1.10)	93%	0.81
Starting and adhering to the effect	11	1.26 (0.98 to 1.62)	100%	0.08

**Table 3:** Summary of Findings (GRADE Assessment).

Outcomes	No. of Participants (Studies)	Certainty of the Evidence (GRADE)	Relative Effect	Anticipated Effects
Risk of All-Cause Dementia	6,325,898 (18 observational studies)	**⨁** **◯◯◯**	RR 1.14	The evidence is very uncertain about the effect of PPIs on dementia risk
		VERY LOW ^1,2^	(0.98 to 1.33)	
Risk of Alzheimer’s Disease	Subgroup analysis	**⨁** **◯◯◯**	RR 1.03	The evidence is very uncertain about the effect of PPIs on Alzheimer’s disease risk.
	(9 observational studies)	VERY LOW ^1,2^	(0.92 to 1.16)	
Dementia Risk in Older Adults (Age ≥ 65)	Subgroup analysis	**⨁⨁**◯◯	RR 1.21	PPI use may slightly increase dementia risk in older adults, but the certainty is low.
	(9 observational studies)	LOW ^2,3^	(1.01 to 1.46)	

**GRADE Working Group grades of evidence High certainty:** We are very confident that the true effect lies close to that of the estimate of the effect. **Moderate certainty**: We are moderately confident in the effect estimate. **Low certainty:** Our confidence in the effect estimate is limited. **Very low certainty**: We have very little confidence in the effect estimate. ^1^ Inconsistency: Downgraded two levels due to critical unexplained heterogeneity (I^2^ = 99%) across the included studies. ^2^ Risk of Bias: Downgraded one level due to serious risk of bias in the majority of included studies (assessed by ROBINS-I), primarily concerning confounding and exposure misclassification. ^3^ Imprecision: Not downgraded. Although the confidence interval is wide, the sample size is large and the lower limit excludes no effect.

## Data Availability

All data, models, and codes generated or used in the study appear in the submitted article.

## Supplementary Materials

The following supporting information can be downloaded at: https://www.mdpi.com/article/10.3390/brainsci16020159/s1. Table S1: The PRISMA checklist. Table S2. Subgroup Analysis and Interaction Test by Geographic Region in Older Adults (≥65 years). Table S3. Results of Sensitivity Analysis for the risk of dementia and PPI use.
